# Crystal structures of human soluble guanylate cyclase catalytic domains: promiscuity of the dimer interface and a potential allosteric site

**DOI:** 10.1186/2050-6511-14-S1-O15

**Published:** 2013-08-29

**Authors:** Opher Gileadi, Charles Allerston, Frank von Delft

**Affiliations:** 1 Structural Genomics Consortium, University of Oxford, Oxford OX3 7DQ, UK

## Background

Soluble guanylate cyclase (sGC) catalyses the synthesis of cyclic GMP in response to nitric oxide. The enzyme is a heterodimer of homologous α and β subunits, each of which is composed of multiple domains.

## Results

We present here crystal structures of a heterodimer of the catalytic domains of the α and β subunits, as well as an inactive homodimer of β subunits. This first structure of a metazoan, heteromeric cyclase provides several observations. First, the structures resemble known structures of adenylate cyclases and other guanylate cyclases in overall fold and in the arrangement of conserved active-site residues, which are contributed by both subunits at the interface. Second, the subunit interaction surface is promiscuous, allowing both homodimeric and heteromeric association; the preference of the full-length enzyme for heterodimer formation must derive from the combined contribution of other interaction interfaces. Third, the heterodimeric structure is in an inactive conformation, but can be superposed onto an active conformation of adenylate cyclase by a structural transition involving a 26º rigid-body rotation of the α subunit. In the modelled active conformation, most active site residues in the subunit interface are precisely aligned with those of adenylate cyclase. Finally, the modelled active conformation also reveals a cavity related to the active site by pseudo-symmetry. The pseudosymmetric site lacks key active site residues, but may bind allosteric regulators in a manner analogous to the binding of forskolin to adenylate cyclase. This indicates the possibility of developing a new class of small-molecule modulators of guanylate cyclase activity targeting the catalytic domain.

**Figure 1:**
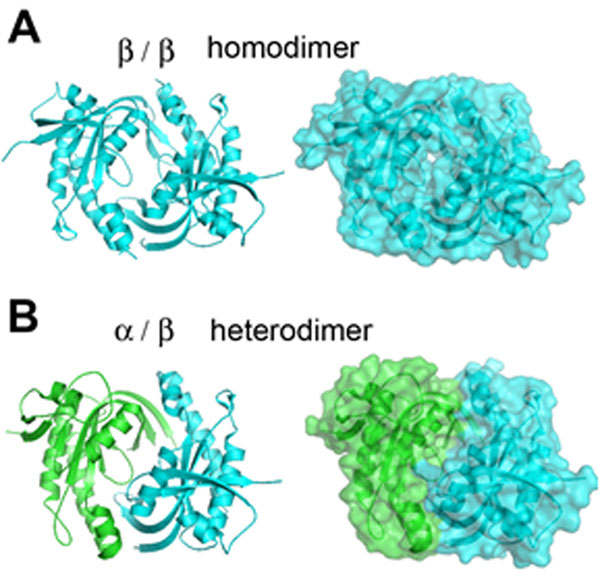
Schematic structures of the catalytic domains of human sGC: A. Homodimer of β subunits, B: Heterodimer of α/β subunits
